# Effects of Converting Tacrolimus Formulation from Twice-Daily to Once-Daily in Liver Transplantation Recipients

**DOI:** 10.1155/2014/265658

**Published:** 2014-07-14

**Authors:** Ashok Thorat, Hong-Shiue Chou, Chen-Fang Lee, Ruey-Shyang Soong, Tsung-Han Wu, Chih-Hsien Cheng, Ting-Jung Wu, Kun-Ming Chan, Wei-Chen Lee

**Affiliations:** ^1^Department of Liver and Transplantation Surgery, Chang-Gung Memorial Hospital, College of Medicine, Chang-Gung University, Taoyuan, Taiwan; ^2^Department of Liver and Transplantation Surgery, Chang-Gung Transplantation Institute, Chang-Gung Memorial Hospital, 5 Fu-Hsing Street, Kwei-Shan, Taoyuan, Taiwan

## Abstract

Typically, tacrolimus is administrated twice daily. Prolonged-release tacrolimus is the once-daily formulation and may be more convenient for patients. Experience with the administration of the once-daily formulation is still limited. This study enrolled 210 liver transplant recipients who had stable liver function and converted tacrolimus from a twice-daily to once-daily formulation on a 1 mg to 1 mg basis. Among 210 patients, seven patients (3.3%) were withdrawn from the once-daily formulation due to allergy and fatigue. For the other patients, the trough concentration after converting to the once-daily formulation was lower than that of the twice-daily formulation. Liver enzymes were mildly elevated in 3 months after formulation conversion and serum creatinine and uric acid were mildly decreased. Seven patients (3.4%) had clinical suspicion of acute rejection after the formulation conversion and three of them were caused by nonadherence. 155 patients (76.4%) experienced a more convenient life with an increase of social activity. Forty-seven patients (23.2%) experienced the convenience of once-daily formulation during overseas trips. In conclusion, tacrolimus can be safely converted from the twice-daily to the once-daily formulation for most stable liver recipients. Acute rejection may occur in a minority of patients during formulation conversion and should be carefully monitored.

## 1. Introduction

Liver transplantation is the only effective treatment for acute or chronic liver failure. Following liver transplantation, acute allograft rejection remains a significant cause of morbidity and may lead to graft dysfunction or failure if it is not treated promptly [1, 2]. By properly conducting immunosuppressive regimens, maintaining immunosuppression, and carefully monitoring drug levels, acute rejection can be successfully prevented or treated. The advances in immunosuppressive agents play an essential role in long-term allograft and patient survival [3, 4]. Cyclosporine, a calcineurin inhibitor, was introduced into transplantation medicine in the late 1970s and helped achieve great successes in solid organ transplantation [5]. Tacrolimus is another potent calcineurin inhibitor introduced in 1989 and becomes one of the most popular immunosuppressive agents in liver transplantation now [6, 7].

Despite the progression of immunosuppressants, acute rejection still occurs in long-term survival patients. This acute rejection may be due to patients' nonadherence to immunosuppressive treatment [2, 8, 9]. The incidence of nonadherence was estimated from 15% to 50% among solid organ transplant recipients [2, 10]. Conventionally, tacrolimus dosage is divided and administered orally every 12 hours to maintain stable trough levels of the drug. The transplant recipients are advised to take tacrolimus on an empty stomach more than one hour before or two to three hours after a meal since oral bioavailability of tacrolimus is variable and absorption of tacrolimus is reduced by food [11, 12]. This requirement may be bothersome to some recipients and thus decrease adherence. Therefore, attempts to reduce the frequency of drug administration may increase adherence to immunosuppressive treatment for transplant recipients.

Prolonged-release tacrolimus has been developed recently and can be administrated once a day [13]. Decrease of dosing frequency usually increases adherence in transplant recipients [8]. Therefore, the once-daily tacrolimus formulation may provide a more convenient regimen than the twice-daily formulation and improve adherence. In preliminary studies with a small group of solid organ transplant recipients, tacrolimus administration could be safely converted from the twice-daily to the once-daily formulation [14–17]. In this study, we evaluated the safety of graft function and looked for additional benefits of converting tacrolimus administration from the twice-daily to the once-daily formulation in a large scale of liver transplant recipients.

## 2. Materials and Methods

### 2.1. Patients

This prospective cohort study was performed to record the safety and tolerability of once-daily tacrolimus administration in stable liver transplant recipients. Patients that were to convert from the twice-daily formulation to the once-daily formulation must have had stable liver function without any acute rejection episodes within three months of entering the study. The study included 210 liver transplant recipients with stable liver function at Chang-Gung Memorial Hospital (Linkou Medical Center). The patients received either deceased or living donor liver transplantation. This study was approved by local ethics committee of Chang-Gung Memorial Hospital (CGMH IRB number 101-2410B).

### 2.2. Tacrolimus Formulation Conversion

Tacrolimus dosage was converted from the regular twice-daily formulation to the prolonged-release once-daily formulation (Astellas Pharma Ltd.) on 1 mg to 1 mg basis. The level of tacrolimus was measured by Flex reagent cartridge (Siemens Healthcare Diagnostics Inc. Newark, DE, USA).

### 2.3. Followup

Liver function, renal function, fasting glucose, uric acid, and trough level of tacrolimus were all measured before and after the conversion of the tacrolimus formulation. After tacrolimus was converted from the twice-daily to the once-daily formulation, all patients were arranged to visit the outpatient clinic one month after conversion for laboratory tests. The patients were advised to call or visit the outpatient clinic at any time if the patients experienced discomfort or any adverse effects that might or might not be related to the conversion of the tacrolimus formulation. All patients were followed up every one to two months if liver function was stable. During followup visits, all patients were closely assessed for adverse events related to the drug, such as rejection, renal toxicity, hepatic dysfunction, diabetes mellitus, gouty arthritis, tremor, and insomnia. In this study, clinical suspicion of acute rejection was defined as more than twice the upper limit of the normal range or an increase of more than 30 U/L over the previous tests after infectious disease and biliary complications were excluded [18]. Liver biopsy was reserved for the patients with persistent abnormal liver function after initial treatments for clinically suspicious acute rejection, biliary complications, or infectious diseases.

### 2.4. Assessment of Social and Travelling Activities

All patients were also asked to personally assess the influence of tacrolimus conversion on daily activities and to score their satisfaction with social and travelling activities. The social satisfaction was scored from 1 to 5: (1) the frequency of social party attendance was less than before; (2) the frequency of social party attendance was the same as before; (3) the frequency of social party attendance was slightly increased; (4) the frequency of social party attendance was much increased; and (5) feel free to join social activities without limitation. The travelling activities were also scored from 1 to 5: (1) decrease travelling activities after formulation conversion; (2) travelling activities were the same as before or after formulation conversion; (3) increase travelling in local areas; (4) increase overnight travelling; and (5) increase overseas travelling. The assessment was performed 9 months after tacrolimus formulation conversion.

### 2.5. Statistical Analysis

All patients were analyzed for efficacy and safety with an intention-to-treat analysis. Paired Student's *t*-test was used to analyze continuous variables. Categorical variables were analyzed by either Chi-square test or Fisher's exact test. All pairwise multiple comparisons were done by Holm-Sidak method. The statistical analyses were all performed with SigmaPlot 12.3 software for Windows (Systat Software, Inc., San Jose, CA, USA). *P* < 0.05 was considered statistically significant.

## 3. Results

### 3.1. Patient Characteristics

This study of tacrolimus formulation conversion included 210 patients, 161 male (76.7%) and 49 female (23.3%). The median (interquartile) age was 52 (45 to 57) years. All the patients were primary recipients of liver transplantation. The median (range) time after liver transplantation for these patients was 55 (9 to 183) months. Among the patients, 134 patients (63.8%) were transplanted for hepatitis B virus- (HBV-) related cirrhosis and 36 (17.1%) patients for hepatitis C virus- (HCV-) related cirrhosis. Most of the patients (*n* = 133, 63.3%) underwent living donor liver transplantation. The details of clinical profiles are listed in Table 1.

#### 3.1.1. Clinical Figures after Tacrolimus Formulation Conversion

Among 210 patients, seven (3.3%) patients were withdrawn from tacrolimus conversion, with four patients experiencing dizziness and fatigue in the morning, two patients experiencing allergic reactions with itchy skin rashes, and one patient experiencing insomnia that affected daily work. For the remaining 203 patients, clinical suspicion of acute rejection was observed in seven patients (3.4%) with elevation of AST and ALT more than 30 U/L from basal lines. The median time of acute rejection attack was three months after formulation conversion with a range from 1.5 to 4.5 months. Among the seven patients, three of their acute rejections (1.5%) were due to nonadherence to the therapy because they forgot to have medicine sometimes. The mean trough levels of tacrolimus for these 7 patients were 6.11 ± 2.02 ng/mL before conversion and decreased to 3.04 ± 1.12 ng/mL when acute rejection was suspected. Four of these rejection episodes were well treated by pulsed therapy of steroids (intravenous injection of 500 mg methylprednisolone) and followed by increasing dosage of the prolonged-release tacrolimus. In the other three patients, the dosage of the prolonged-release tacrolimus was modestly increased to keep a higher trough level and liver function returned within normal limits. All these acute rejection episodes were well response to steroid injection or increasing doses of tacrolimus; therefore, no liver biopsy was performed. For the other patients without rejection, the once-daily administration of tacrolimus was well tolerated and demonstrated easy handling with less stress than the mandatory and scheduled intake of the twice-daily formulation.

### 3.2. Dosage and Blood Levels of Tacrolimus

**Figure 1:**
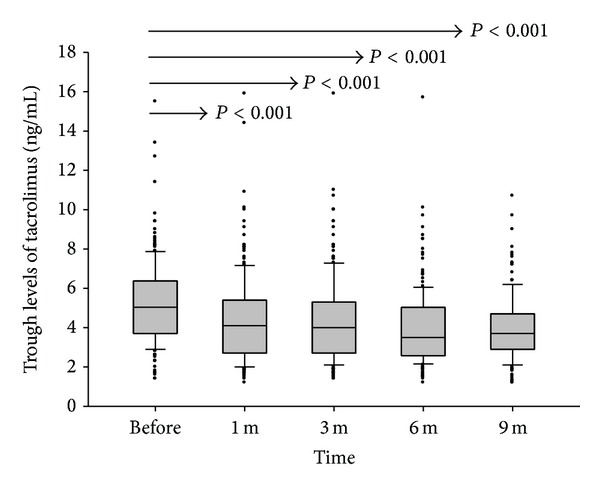
Trough levels of tacrolimus before and after formulation conversion. Based on 1 mg to 1 mg conversion, the trough levels of tacrolimus at 1, 3, 6, and 9 months after formulation conversion were all significantly lower than that before conversion.

The conversion of tacrolimus from the twice-daily to the once-daily formulation was based on 1 : 1 mg. The median (interquartile) total daily dose of tacrolimus before conversion was 3 (2 to 4) mg with a range from 1 to 9 mg. Trough levels of tacrolimus just before conversion and in 1, 3, 6, and 9 months after conversion were recorded for comparison. Before conversion, the mean trough level of tacrolimus was 5.26 ± 2.12 ng/mL. After one month of tacrolimus formulation conversion, the mean trough level of tacrolimus was 4.38 ± 2.21 ng/mL, which was significantly lower than that before conversion (*P* < 0.001). In the following months, the trough levels of tacrolimus were kept at 4.33 ± 2.18 ng/mL, 4.00 ± 1.91 ng/mL, and 4.00 ± 1.63 ng/mL at the 3, 6, and 9 months, respectively (Figure 1).

### 3.3. Liver and Renal Functions

**Figure 2:**
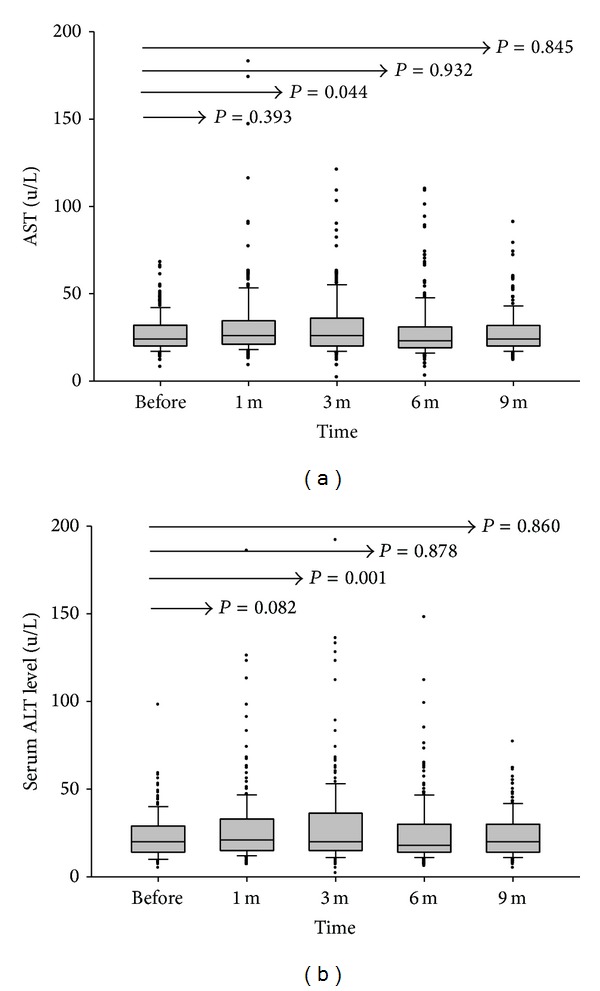
The serum levels of AST and ALT before and after formulation conversion. (a) The median (interquartile) level of AST was elevated from 24 (20 to32) U/L to 26 (21.0 to 34.5) U/L at 1 month (*P* = 0.393), reached its peak (26 (20 to 36) U/L) at 3 months (*P* = 0.044), and returned to 23 (19 to 31) U/L at 6 months (*P* = 0.932) and 24 (20 to 32) U/L at 9 months (*P* = 0.845). (b) The median (interquartile) level of ALT was elevated from 20 (14 to 29) U/L to 21 (15 to 33) U/L at 1 month (*P* = 0.082), reached its peak (20 (15 to 36) U/L) at 3 months (*P* = 0.001), and returned to 18 (14 to 30) U/L at 6 months (*P* = 0.878) and 20 (14 to 30) U/L at 9 months (*P* = 0.860).

**Figure 3:**
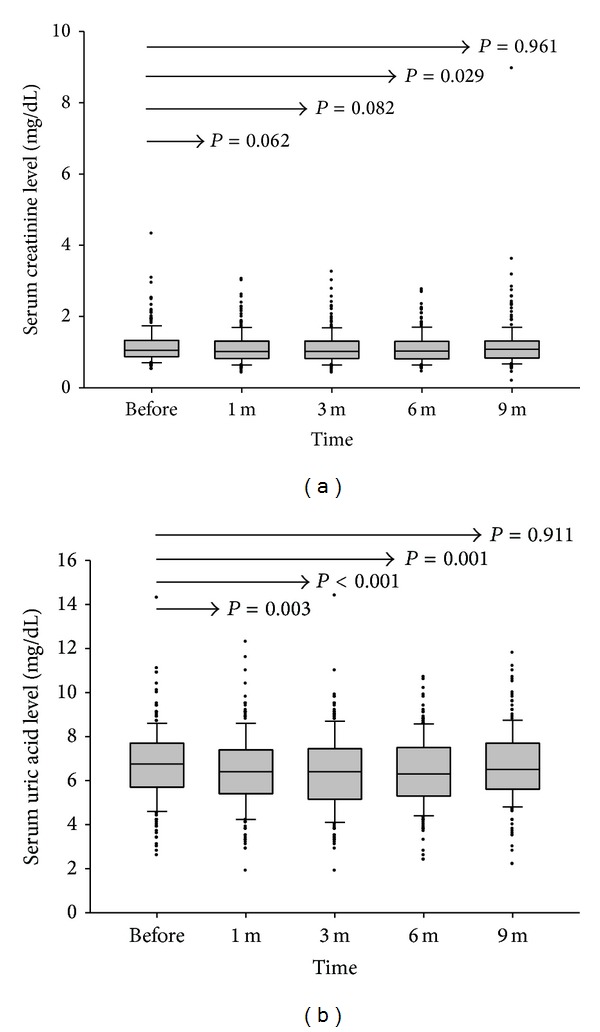
The serum levels of creatinine and uric acid before and after formulation conversion. (a) The median (interquartile) level of creatinine was decreased from 1.050 (0.870–1.330) mg/mL to 1.015 (0.820–1.310) mg/mL at 1 month (*P* = 0.062), 1.020 (0.820–1.310) mg/mL at 3 months (*P* = 0.082), and 1.030 (0.810–1.300) mg/mL at 6 months (*P* = 0.029). It increased to 1.080 (0.835–1.315) mg/mL again at 9 months (*P* = 0.961). (b) The median (interquartile) level of uric acid was decreased from 6.75 (5.70–7.70) mg/mL to 6.40 (5.40–7.40) at 1 month (*P* = 0.003), 6.40 (5.15–7.45) mg/mL at 3 months (*P* < 0.001), and 6.30 (5.30–7.50) mg/mL at 6 months (*P* = 0.001). It increased to 6.50 (5.60–7.70) mg/mL again at 9 months (*P* = 0.911).

Liver, renal, and metabolic functions were assessed before and after tacrolimus conversion. In the first month after tacrolimus formulation conversion, liver function did not differ significantly. However, aspartate aminotransferase (AST) and alanine aminotransferase (ALT) elevated in the third month. The median (interquartile) level of AST elevated from 24 (20 to 32) U/L before conversion to 26 (20 to 36) U/L at the 3 months (*P* = 0.044), and ALT was elevated from 20 (14 to 29) U/L to 20 (15 to 36.3) U/L (*P* = 0.001). At 6 months, the median (interquartile) levels of AST and ALT returned to 23 (19 to 31) U/L and 18 (14 to 30) U/L, respectively, which were similar to levels before conversion (*P* = 0.932 and 0.878, resp.) (Figures 2(a) and 2(b)). Although AST and ALT were increased slightly in the first 3 months, they were still within the normal limit and did not cause symptoms and signs, aside from the seven patients that experienced acute rejection. In terms of renal function, the median (interquartile) serum creatinine level was 1.05 (0.87–1.33) mg/L before conversion and improved to 1.03 (0.82–1.31) mg/L at 6 months (*P* = 0.029) and went back to 1.08 (0.835–1.315) mg/L at 9 months (*P* = 0.961) (Figure 3(a)). The levels of uric acid improved in the first 6 months and returned to the original level after 9 months (Figure 3(b)). The levels of fasting sugar did not change throughout the full course. All the patients have been followed up for more than 9 months until now. The liver function, renal function, fasting sugar, and uric acid are almost at the same levels before conversion.

### 3.4. Adverse Effects for Once-Daily Tacrolimus

The adverse effects of prolonged-release tacrolimus were considered. After tacrolimus was converted to the once-daily formulation, only a few patients had adverse effects. In addition to the seven patients withdrawn from the once-daily formulation, two patients (1.0%) had new-onset of hypertension and needed medication, two patients (1.0%) felt increasing frequency of headache, and one patient had increasing frequency of tremors after taking the prolonged-release tacrolimus.

#### 3.4.1. Social Activity

**Figure 4:**
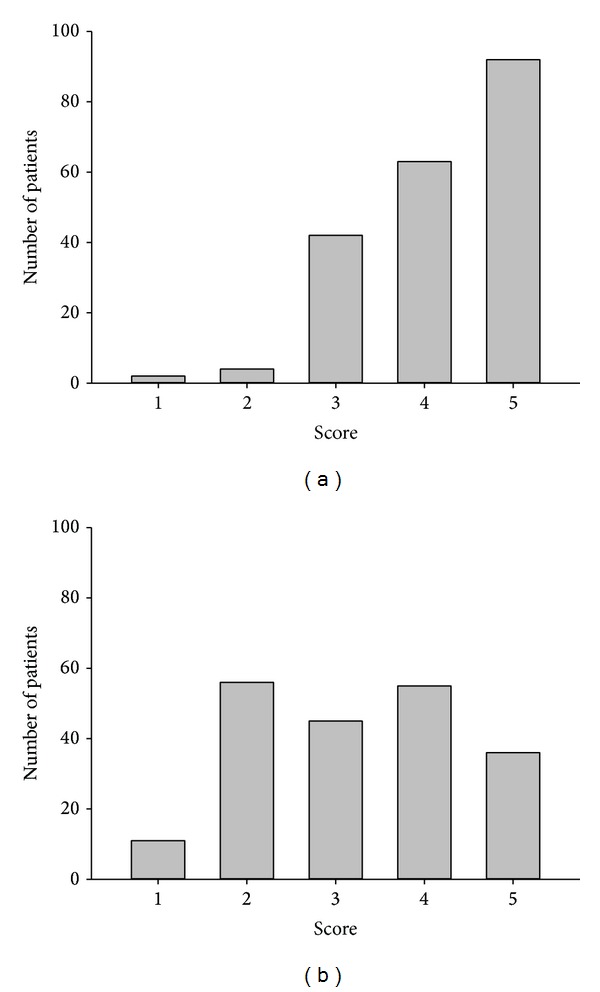
The patient distribution of satisfactory scores for social and travelling activities. (a) Patient personally scored their satisfaction toward social activity on a scale from 1 to 5, each number, respectively, representing poor, fair, good, very good, and excellent. 155 patients (76.4%) scored 4 or 5 in their satisfaction toward social activity. (b) The increase of travelling activity was also scored from 1 to 5. 91 patients (44.8%) scored 4 or 5 in their increase of travelling activities.

In this study of formulation conversion, we paid special attention to the social and travelling activities of the patients after tacrolimus was converted from the twice-daily to the once-daily formulation. Because dinner party and travelling together were the most common and important daily social activities for Taiwanese, the designed scores were according to the frequency of party join and travelling activities. The patients were asked to personally score their satisfaction with social activities from 1 to 5 as described in method section. For all the patients, the satisfaction score of social activities was 4.17 ± 0.92. 155 of 203 patients (76.4%) experienced satisfied social activity (social activity score ≥ 4) because they could join dinner parties without worrying about the administration of tacrolimus (Figure 4(a)). In terms of travelling, 91 patients (44.8%) increased their overnight or overseas travelling activities while 112 patients did not. The score for travelling activities was 3.21 ± 1.27. 47 patients (23.2%) went on long or overseas trips and expressed the convenience of the once-daily tacrolimus administration due to the lack of confusion over tacrolimus administration across different time zones (Figure 4(b)). Once-daily tacrolimus allowed our patients to freely join dinner parties and was convenient for the patients who travelled across different time zones.

## 4. Discussion

This study demonstrates that the twice-daily tacrolimus formulation can be converted to the once-daily tacrolimus formulation safely in most liver transplant recipients with stable liver function. Among the 210 patients who underwent the tacrolimus formulation conversion, only seven patients were converted back to the twice-daily formulation due to allergy or fatigue. According to drug development, only the matrix system of the drug was altered, and the drug was modified to prolong releasing [12]. However, some patients might be allergic to the matrix system of prolonged-release in this study. According to pharmacokinetics, 24-hour drug exposure for standard and prolonged-release tacrolimus is similar [12]. However, the incidence of fatigue was higher for the once-daily formulation than for the twice-daily formulation [13]. The mechanism of fatigue was not really known and needed further investigation. Although several patients were discomforted and converted back to standard tacrolimus, conversion of tacrolimus from the twice-daily to the once-daily formulation succeeded in the majority of liver transplant recipients.

The blood trough concentration of tacrolimus was decreased and liver enzymes were mildly elevated 3 months after the formulation was converted from the twice-daily to the once-daily tacrolimus. In this study, the dose of tacrolimus conversion was based on 1 mg to 1 mg. The trough concentrations of tacrolimus recorded at 1, 3, 6, and 9 months after conversion were all lower than the concentration before formulation conversion. The trough concentration of tacrolimus for our patients was only 5.26 ± 2.12 ng/mL before conversion. Further decreased trough concentration of tacrolimus after formulation conversion caused mild elevation of liver enzymes, AST and ALT, which was not seen in the first month but was seen in the third month. Although the serum levels of AST and ALT were elevated, they were still within the normal limits and returned to the initial basal levels without increasing the doses of tacrolimus later on. It was already known that the trough concentration of the once-daily formulation was lower than that of the twice-daily formulation based on 1 mg to 1 mg conversion [14, 19]. In the* de novo* study, a higher dose of once-daily than twice-daily formulation was suggested to keep the same trough concentration of tacrolimus [20]. Therefore, if the formulation conversion to once-daily tacrolimus on 1 mg to 1 mg basis was adopted for stable liver transplant recipients, a lower trough concentration of tacrolimus could be expected. The elevation of AST and ALT should be cautiously monitored, which may not occur immediately after formulation was converted but rather occur within the first three months of the formulation conversion.

Acute rejection might occur in stable liver transplant recipients after formulation conversion on the 1 mg to 1 mg basis. In this study, seven patients had clinical suspicion of acute rejection after formulation conversion. Three of the patients clearly mentioned that they forgot to take medication sometimes while the other four patients denied nonadherence to the medication advice. While the formulation was converted from the twice-daily to the once-daily formulation, it was thought that adherence of medication would be increased. However, there was no evidence to prove that adherence was increased in this study. Contrarily, if patients neglected to take the medication, a daily dosage of tacrolimus would be completely missed. In this study, three patients neglected to take tacrolimus, and acute rejection occurred between 2 and 4.5 months after the formulation conversion. Of patients with good adherence, four patients had acute rejection due to a lower trough concentration of tacrolimus, which occurred between 1.5 and 4 months after the formulation conversion. All these episodes of acute rejection were easily treated by steroid injection or increased dosage of the once-daily tacrolimus. These clinical observations revealed that acute rejection was easy to occur if patients neglected to take the medication. While the formulation was successfully converted to the once-daily tacrolimus, acute rejection may occur one and a half months after the formulation conversion due to a lower trough concentration.

Some clinical profiles were also altered after the tacrolimus formulation conversion. The serum levels of creatinine and uric acid were decreased after conversion from the twice-daily to the once-daily formulation. The clinical data clearly showed that the serum levels of creatinine were decreased in the first 6 months after the formulation conversion. The serum level of uric acid also decreased in the first 6 months. However, these differences in the levels of creatinine and uric acid disappeared 9 months after conversion. The decreased levels of creatinine and uric acid might be due to a lower trough level of tacrolimus after converting to the once-daily tacrolimus formulation. As the trough concentration of tacrolimus was steady after formulation conversion, we could not clearly understand why the creatinine and uric acid differences disappeared after 9 months of conversion; further study is needed. Tacrolimus has also been reported to induce mellitus diabetes or glucose intolerance. In this study, the fasting sugar was not changed before or after tacrolimus formulation conversion.

The conversion of tacrolimus from the twice-daily to the once-daily formulation increased the social activity of our patients. Allowing patients to regain social activity is an important goal after liver transplantation. Social activities were markedly increased in 76.4% of our patients after tacrolimus was converted from the twice-daily to the once-daily formulation. Satisfactory scores personally assessed by the patients were as high as 4.17 ± 0.92 (5 at maximum). Oral bioavailability of tacrolimus varies among transplant patients. The decrease of bioavailability is greater for Asian, African American, and Hispanic patients than for Caucasian patients [13]. Administration of tacrolimus requires an empty stomach to increase absorption of the drug. The patients are required to fast at least one hour before and after taking tacrolimus. This requirement was always troublesome for our patients who partook in dinner parties, which are the most important social activity for Taiwanese and Asian people. During dinner parties, patients face the dilemma of fasting or, against medical advice, partake in the meal. Gradually, patients that face such a decision may withdraw from social activities. Yet, the once-daily formulation of tacrolimus changed the situation. The once-daily formulation allows patients to only take the medication in the morning, thus enabling them to join dinner parties or any kind of social activity in the evening more freely. Therefore, most of our patients were satisfied with the once-daily formulation since their daily lives were not interrupted by immunosuppressive agents.

Another benefit toward the lives of our patients was that the once-daily formulation was convenient for patients who liked travelling, especially patients who would travel across different time zones. Although travelling did not increase among all of our patients, 47 patients who went on long, overseas trips enjoyed the convenience of the once-daily formulation since they were no longer confused as to when to take the formulation. This convenience was most apparent when patients travelled between different time zones. The only requirement was that the patients simply remembered to take medication every 24 hours. Thus, it was very easy to calculate the exact time to take tacrolimus in a different time zone.

## 5. Conclusion

Conversion of tacrolimus from the twice-daily to the once-daily formulation was safe for most liver transplant recipients with stable liver function. Based on 1 mg to 1 mg conversion, the trough concentration of the once-daily tacrolimus was lower than that of the twice-daily formulation, AST and ALT were mildly elevated, and acute rejection should be cautiously monitored. The bonus of tacrolimus converted to once-daily formulation is the increase of social activity for our patients and convenience when travelling across different time zones.

## Figures and Tables

**Table 1:** The clinical characteristics of 210 patients with conversion of tacrolimus from twice-daily to once-daily formulation.

Male	161 (76.7%)
Age (years)	52 (45–57) [4–69]∗
Etiologies of liver transplantation	
Hepatitis B	134 (63.8%)
Hepatitis C	36 (17.1%)
Hepatitis B + C	9 (4.3%)
Alcoholic	11 (5.2%)
Others	20 (9.5%)
HCC (+)	85 (40.5%)
Type of transplantation	
Whole liver	53 (25.2%)
Split liver	24 (11.4%)
Living donor	133 (63.3%)
Tacrolimus dosage (mg/day) before conversion	3 (2–4)[1–9]∗

*Median (interquartile) [range].

## Abbreviations

ALT: Alanine aminotransferase
AST: Aspartate aminotransferase
HBV: Hepatitis B virus
HCV: Hepatitis C virus.
